# County‐level correlates of dental service utilization for low income pregnant women. Ecologic study of the North Carolina Medicaid for Pregnant Women (MPW) program

**DOI:** 10.1186/s12913-021-06060-9

**Published:** 2021-01-13

**Authors:** Mark E. Moss, Andrew Grodner, Ananda P. Dasanayake, Cherry M. Beasley

**Affiliations:** 1 Department of Foundational Sciences, School of Dental Medicine, East Carolina University, Greenville, USA; 2 Department of Economics, East Carolina University, North Carolina Greenville, USA; 3 Department of Epidemiology and Health Promotion, College of Dentistry, New York University, New York, USA; 4 Department of Nursing, University of North Carolina – Pembroke, Pembroke, USA

**Keywords:** Pregnancy, Dental care, Medicaid, Health Policy

## Abstract

**Background:**

Dental care utilization for low income pregnant women is met with challenges in the traditional dentist-centered model of care. County-level measures provide insights for policy and roles for stakeholders that extend beyond the dentist-patient relationship. We examined county-level data to generate hypotheses about factors that influence utilization of dental services in North Carolina’s Medicaid for Pregnant Women (MPW) program.

**Methods:**

County-level Medicaid utilization data for dental services for 2014–2016 were pooled to get mean county estimates of dental utilization in the MPW program. Descriptive statistics and multivariate regression models of dental utilization and county-level measures are presented. Data used were collected by NC Child and the Robert Wood Johnson Foundation’s County Health Rankings Reports. USDA Economic Research Service data were used to categorize counties in terms of Farming, Recreation, Persistent Poverty, and metro/non-metro status using Rural Urban Continuum Codes.

**Results:**

Dental utilization ranged from 1–26% with a median of 8.5% across the 100 counties of North Carolina. Strong patterns linking utilization of dental services in the MPW program to contextual social measures of well-being emerged, specifically, increased reporting of child abuse and neglect, elevated infant mortality, poor quality of life, and worse ranking in years of potential life lost. Counties with persistent poverty had lower rates of dental utilization.

**Conclusions:**

Utilization of dental services in the MPW program is generally low. Patterns identify the potential for enhancing community-clinical linkages to improve birth outcomes and care coordination for pregnant women to enhance dental utilization in this population.

Dental coverage in the Medicaid program in most states is administered separately from medical coverage. The separation of the funding mechanisms adds a further layer of complexity to care integration. Efforts to enhance dental care for pregnant women in the Medicaid program may benefit from policy that aligns incentives for care coordination within the community. Policy that extends the window of eligibility for dental benefits to 24 months after the birth of the child will help women complete the dental treatment that is needed. This also leverages the value of care coordination for community stakeholders from diverse child health sectors.

## Background

The importance of oral health during pregnancy is often overlooked and this has been framed as a “call to action”[1]. Significant opportunities for interprofessional care exist [2–4] and population health tools are emerging that can be tailored to address these gaps [5]. The patient-centered (or person-centered) medical home (PCMH) is an integrated approach to providing comprehensive primary care for children, adolescents, and adults. PCMH is a whole-person oriented approach to health care. In the context of pregnancy, PCMH implies an approach where perinatal health care partnerships are formed among clients, other health professionals including dental health professionals, and social support systems to foster optimal perinatal health outcomes [6,7]. Care coordination for low income pregnant women has been shown to be effective in improving pregnancy outcomes [8].

The term Pregnancy Medical Home has been used to describe community-based care for pregnant women in North Carolina (NC). It has emerged as an effective model for care coordination among pregnant women who are covered by the NC Medicaid program [9, 10]. It started in 2011 and has expanded to 94 of North Carolina’s 100 counties. It is a community-level partnership that aims to reduce infant mortality using case management and care coordination that targets high risk pregnancies. Pregnancy care management is delivered by registered nurses and social workers both regionally and within 84 county health departments across the state. There are no financial incentives for dental care services directly tied to the Pregnancy Medical Home initiative. This creates further barriers for dental utilization.

In NC, dental benefits are available for low income women who do not meet the standard income eligibility levels for Medicaid while they are pregnant through the Medicaid for Pregnant Women (MPW) program. The MPW program is designed to help low income pregnant women get access to health care by loosening the income requirements so that women with incomes as high as 185% of FPL are eligible. It is a program with unique challenges for women who do not have a usual source of care for dental services. The dental benefit for the pregnant woman in the MPW program ends when the baby is born.

Value can arise from community-level systems and supports [11] that influence utilization of dental care services by low-income pregnant women. In this paper we use county-level measures to generate hypotheses about conditions and policies that may influence community-clinical linkages that improve utilization of dental services by pregnant women in the MPW program in NC.

## Methods

County-level data for this study came from several sources. None of the sources had individual/personal identifiers. The dependent variable came from NC Medicaid claims filed for the MPW program in each of three fiscal years 2014–2016. County status was the place of residence of the recipient (rather than provider place of service). Dental service utilization represented any dental service that was filed as a claim under the MPW program in the NC Medicaid system.

Independent variables were organized around Andersen’s Model for assessing health care utilization to include Predisposing Factors, Enabling Factors, Indicators of Need, and Health Services measures [12]. Predisposing factors were taken from county classifications developed by the United States Department of Agriculture Economic Research Service. Indicators of need were not specific for dental problems but were taken from a broad range of measures collected by NC Child and The Robert Wood Johnson Foundation’s County Health Rankings to reflect the well-being of the maternal and child population in a county. High school graduation rate was taken from the County Health Rankings data but considered a Predisposing Factor. Dental health service indicators were number of dentists per 100,000 population in a county and whether a county had a Federally Qualified Health Center (FQHC) that provided dental services.

The Rural Urban Continuum Code (RUCC) system was used to characterize metro and non-metro counties [13]. Codes range from 1 to 9. This is a classification that attempts to arrange counties on a continuum from most metro to least metro based on population density and proximity of non-metro counties to urban areas.

County Typology Codes identified counties with Persistent Poverty and the dominant economic driver in the county as Farming, Recreation, Manufacturing, Government, or Non-specialized [14]. Persistent Poverty is a county-level designation that identifies counties where at least 20 percent of the population is at or below the Federal Poverty Level in each of the census years of 1980, 1990, 2000 and the American Community Survey of 2013. In North Carolina, 10 counties are designated as areas of Persistent Poverty. Persistent Poverty is often a marker that alerts providers of direct and indirect services to the increased vulnerability of the population especially low educational attainment and poor health outcomes.

Analytic Approach.

In this study we used both univariate and multivariate statistical techniques. The univariate descriptive measures make use of spatial visualization of the dental utilization rate with map procedure provided by SAS version 9.4, as well as basic descriptive statistics. We employed multivariate regression modeling to analyze the conditional impact of covariates on the dental utilization rate. The primary selection criteria for the best model was statistical significance of the coefficients and fit as measured by the r-square and adjusted r-square.

Our primary dependent variable is percent utilization rate (PUR) defined as the proportion of eligible women in the MPW program who had a claim for any dental service. We used three years for our study, 2014–2016, which we combined into a single sample of 100 observations by calculating PUR as the ratio between total number of mother-births utilizing dental services and total number of eligible mother-births over the three-year period for a county. Use of a three-year summary value provided some stability to the data since some counties had fewer than 100 eligible women in a given year.

We used Andersen’s Behavioral Model to frame variables related to health services utilization [12], where the key Enabling factor is financial access through the MPW program. Pre-disposing factors for a county are represented as Economic indicators, labeled as Group 1, and measures of Need for a county are Social Well Being indicators, labeled as Group 2. Health care system factors are Dental Workforce indicators, labeled as Group 3. The Economic indicators in Group 1 included Farming/Recreation classification, Persistent Poverty, and High School Graduation rate. The Social Well Being indicators of Group 2 were Child Abuse and Neglect Reporting in 2015, Change in abuse and neglect reporting, Infant Mortality, Length of Life rank, and county-level average of self-reported Poor Mental health days in the past 30 days. The Dental Workforce indicators were number of dentists per 100,000 population and whether or not a Federally Qualified Health Center that provided dental services was located in the county.

## Results

Dental utilization in the MPW program ranged from 1 percent to 26 percent with a median of 8.5 percent. A major focus of this study was to determine whether characterization of the context of rurality identifies differences in health utilization rates for dental services. Therefore, we began by presenting basic statistics in Table 1. It is notable that there is no clear pattern when it comes to PUR by RUCC, except for the fact that the last category has the highest level of variability, (RUCC = 9) rural areas not adjacent to a metro area. We note that all 95% confidence intervals overlap indicating that statistically, there are no pairwise mean differences.

**Table 1:** Percent Utilization Rate (PUR) for dental services by eligible women in the Medicaid for Pregnant Women Program by RUCC for North Carolina Counties 2014–2016

Rural Urban Continuum Code Description	Mean PUR	Std	N (counties)	Min	Max	95% Confidence Interval	
(1) Metro areas of 1 million or more	9.06	2.61	12	4.79	15.21	(7.40	10.72)
(2) Metro areas of 250,000 to 1 million	8.56	2.57	25	4.57	13.60	(7.50	9.62)
(3) Metro areas of fewer than 250,000	10.07	3.10	9	7.02	15.57	(7.69	12.46)
(4) Urban areas of 20,000 or more, adjacent to metro	9.83	4.33	15	4.72	18.65	(7.43	12.23)
(5) Urban areas of 20,000 or more, not adjacent to metro	8.05	3.32	2	5.70	10.39	(-21.73	37.83)
(6) Urban areas of 2,500 to 19,999, adjacent to metro	8.99	2.78	16	4.53	14.56	(7.51	10.47)
(7) Urban areas of 2,500 to 19,000, not adjacent to metro	8.83	4.54	5	5.82	16.71	(3.19	14.47)
(8) Rural areas of or less than 2,500, adjacent to metro	8.22	3.21	9	3.39	13.50	(5.57	10.69)
(9) Rural areas of or less than 2,500, not adjacent to metro	13.18	8.58	7	0.97	26.19	(5.24	21.11)

Note: RUCC is Rural Urban Continuum Code (USDA)

**Fig. 1:**
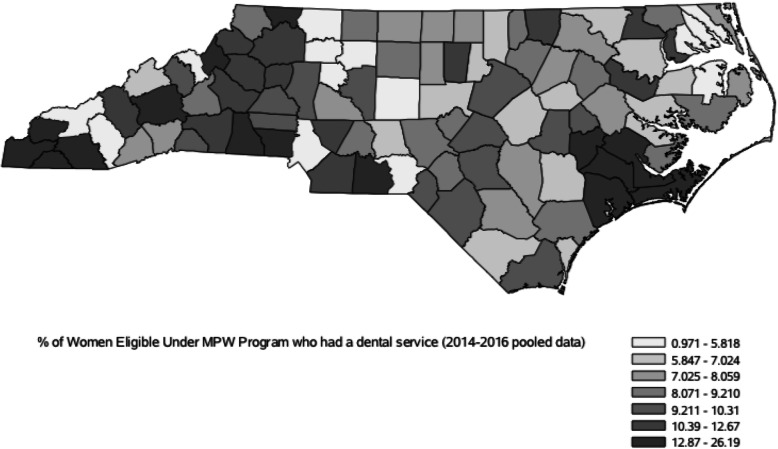
County-level percent of MPW eligible women who had any dental service in North Carolina (pooled 2014–2016)

Figure 1 presents an overall spatial pattern of PUR in the state of North Carolina using a map of counties. It is notable that the spatial distribution is not random, with clusters of relatively high utilization rate in the western and south eastern parts of the state. On the other hand, central and north eastern counties have lower percent utilization rates with few exceptions.

**Fig. 2:**
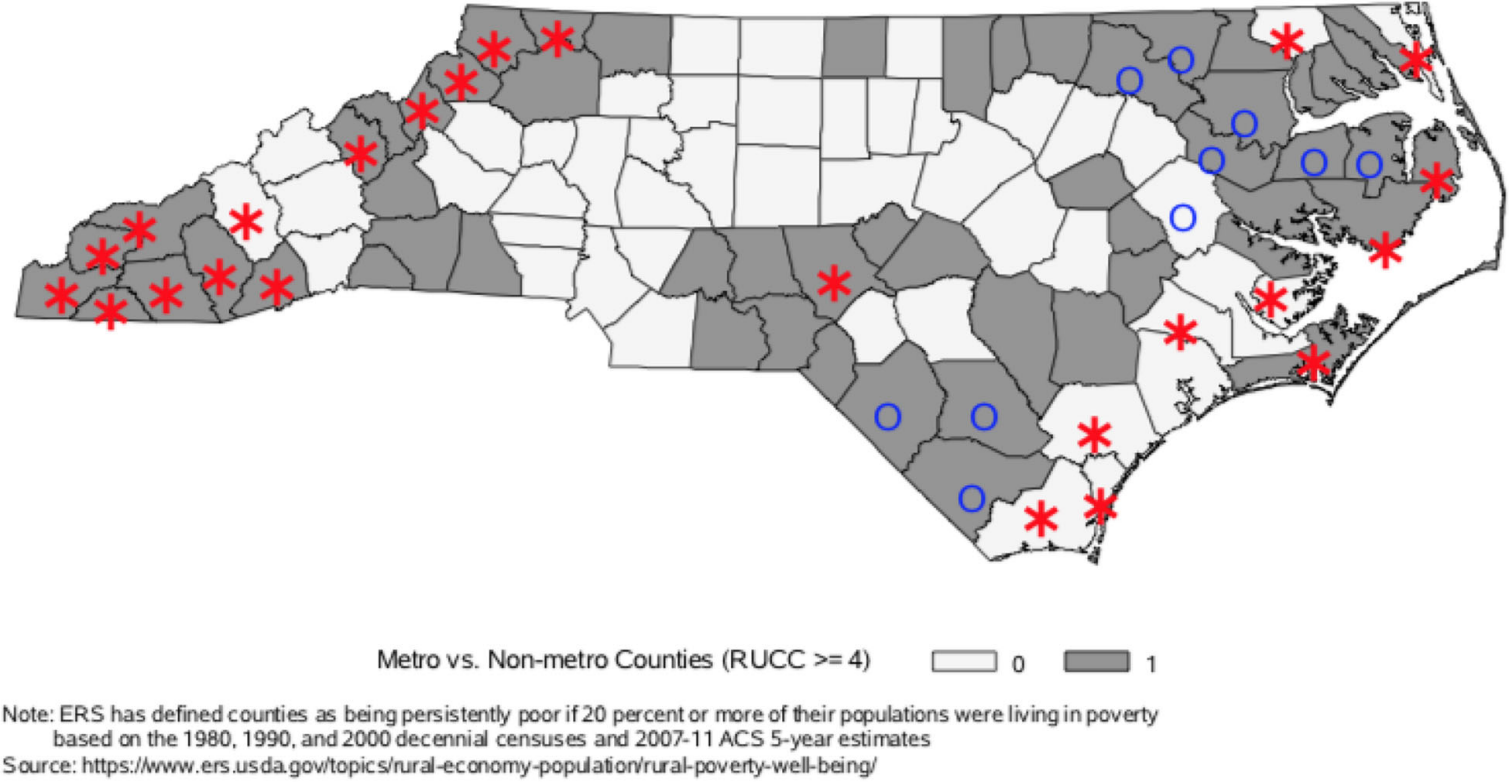
Metro vs. Non-metro Counties (RUCC classification 4–9) in North Carolina with counties classified as Farming or Recreation (2015), marked as red* and counties classified as persistently poor (2013), marked as blue O

In order to have a better understanding of RUCC we also present a map showing the spatial distribution of this classification in the state (Fig. 2). We divided the sample into 2 groups based on metro status with Metro Counties (RUCC < = 3) and Non-metro counties (RUCC > = 4). Such classification is warranted because the RUCC scheme is intended to highlight proximity to services that are generally concentrated in metro areas.

Descriptive statistics for variables used in our analysis are presented in Table 2. Note that not all variables are available in all 100 counties. There are two observations missing for High School Graduation rate and Poor Mental Health days. These counties have small populations and the data sources did not provide values for them. Even though those missing values limit our sample, sensitivity analysis revealed that our baseline results remain essentially the same and there is no systematic pattern for those omitted observations reducing risk of bias in our statistical analysis.

**Table 2:** Descriptive Statistics in Full Sample (all counties) and by Non-Metro and Metro classification

Variable	Mean	Std	N	Min	Max	95% Confidence Interval	
**FULL SAMPLE**							
Percent Utilization Rate (PUR) dental care	9.31	3.83	100	0.97	26.19	8.55	10.07
Rural Urban Continuum Code (RUCC)	4.25	2.54	100	1	9	3.74	4.75
**Group 1 Predisposing Factors**							
Farming/Recreation	0.24	0.43	100	0	1	0.15	0.32
Persistent Poverty	0.10	0.30	100	0	1	0.04	0.16
High School Graduation Rate	81.59	4.97	98	68.00	92.50	80.59	82.58
**Group 2 County Well-being**							
Abuse reporting past year	63.68	23.57	100	18.75	157.86	59.00	68.35
Change in Abuse reporting	-1.40	10.77	100	-52.03	40.46	-3.54	0.73
Infant Mortality	7.83	4.76	100	0.00	24.10	6.88	8.77
Length of Life County Rank	50.50	29.01	100	1	100	44.74	56.26
Poor Mental Health Days in past month	3.65	0.86	93	2.00	6.20	3.47	3.82
**Group 3 Dental Health Services**							
Dentists per 100,000 pop	36.27	24.12	100	0	184.54	31.48	41.06
FQHC Dental in County	0.28	0.45	100	0	1	0.19	0.37
**NON-METRO COUNTIES (RUCC > = 4)**							
Percent Utilization Rate (PUR) dental care	9.59	4.59	54	0.97	26.19	8.33	10.84
Rural Urban Continuum Code (RUCC)	6.22	1.76	54	4	9	5.74	6.7
**Group 1 Predisposing Factors**							
Farming/Recreation	0.30	0.46	54	0	1	0.17	0.42
Persistent Poverty	0.17	0.38	54	0	1	0.06	0.26
High School Graduation Rate	80.99	5.46	52	68.00	92.50	79.47	82.51
**Group 2 County Well-being**							
Abuse reporting past year	67.38	27.22	54	18.75	157.86	59.95	74.8
Change in Abuse reporting	-1.64	13.50	54	-52.03	40.46	-5.33	2.04
Infant Mortality	8.34	5.88	54	0.00	24.10	6.73	9.94
Length of Life County Rank	59.87	28.54	54	3	100	52.08	67.66
Poor Mental Health Days in past month	3.66	0.93	50	2.00	5.70	3.39	3.92
**Group 3 Dental Health Services**							
Dentists per 100,000 pop	31.03	16.35	54	0	70.14	26.57	35.49
FQHC Dental in County	0.26	0.44	54	0	1	0.14	0.38
**METRO COUNTIES (RUCC < = 3)**							
Percent Utilization Rate (PUR) dental care	8.99	2.69	46	4.57	15.5689	8.18	9.78
Rural Urban Continuum Code (RUCC)	1.93	0.68	46	1	3	1.73	2.13
**Group 1 Predisposing Factors**							
Farming/Recreation	0.17	0.38	46	0	1	0.06	0.29
Persistent Poverty	0.02	0.15	46	0	1	-0.02	0.07
High School Graduation Rate	82.27	4.32	46	72.35	90.20	80.98	83.55
**Group 2 County Well-being**							
Abuse reporting past year	59.34	17.71	46	31.18	107.67	54.08	64.6
Change in Abuse reporting	-1.12	6.35	46	-22.45	12.35	-3.00	0.76
Infant Mortality	7.23	2.90	46	0.00	12.40	6.37	8.09
Length of Life County Rank	39.50	25.76	46	1	90	31.85	47.15
Poor Mental Health Days in past month	3.65	0.79	43	2.10	6.20	3.40	3.89
**Group 3 Dental Health Services**							
Dentists per 100,000 pop	42.42	29.89	46	0	184.54	33.54	51.3
FQHC Dental in County	0.30	0.47	46	0	1	0.17	0.44

Notes on Variables:

*County Codes from US Department of Agriculture (USDA)*

RUCC is the Rural Urban Continuum Code system.^8^ Codes range from 1 to 9. This is a classification scheme developed by the USDA. It attempts to arrange counties on a continuum from most metro to least metro based on population density and proximity of non-metro counties to urban areas

County Typology Codes^9^ for Persistent Poverty, Farming, Recreation

Persistent Poverty is a county-level designation that identifies counties where at least 20 percent of the population is at or below the Federal Poverty Level in each of the census years of 1980, 1990, 2000 and the American Community Survey of 2013. In North Carolina, 10 counties are designated as areas of Persistent Poverty. Persistent Poverty is often a marker that alerts providers of direct and indirect services to the increased vulnerability of the population especially low educational attainment and poor health outcomes

Farming/Recreation is derived to represent counties that were designated as either Farming or Recreation. Farming is the Farm-dependent county indicator, where 0 = no 1 = yes. Farming accounted for at 25% or more of the county’s earnings or 16% or more of the employment averaged over 2010–2012

Recreation defines counties (0 = no 1 = yes) based on computation from three data sources: (1) Percentage of wage and salary employment in entertainment and recreation, accommodations, eating and drinking places, and real estate as a percentage of all employment reported by the Bureau of Economic Analysis; (2) Percentage of total personal income reported for these same categories by the Bureau of Economic Analysis; and (3) Percentage of vacant housing units intended for seasonal or occasional use reported in the 2010 Census. The three variables measuring employment, earnings, and seasonal housing were converted to z-scores and combined into a weighted index (weights of 0.3 were assigned to income and employment and 0.4 to seasonal housing) to reflect recreational activity. Counties with index scores of 0.67 or higher were regarded as recreation counties. Seasonal housing was given a higher weight because in some areas employment and income may not reflect recreational activity because of the seasonality. The comparison group was all other counties and these were designated as either Manufacturing, Government, or Non-specialized

*Variables from NC Child (*http://www.ncchild.org/*)*

Abuse and Neglect claims in 2015 and 2016. The rate per 1000 of children under age 18 who were assessed for abuse or neglect

Infant Mortality in 2015. This represents the number of infant deaths per 1000 babies born alive

*North Carolina County Health Rankings 2015 variables* (http://www.countyhealthrankings.org/rankings/data/NC)

Length of Life – County rank among counties in North Carolina (from 1 to 100, with 100 being worst) for age-adjusted Years of Potential Life Lost before age 75, calculated from 2010–2012 Mortality files National Center for Health Statistics

Quality of Life - from BRFSS 2006–2012, measured as county-level average number of mentally unhealthy days (poor mental health days) in the past 30 days (age-adjusted). Poor mental health days ranged from 2.0 to 6.2 days per month, median of 3.6

High School Graduation Rate = (percent) number students that graduated divided by the number of students expected to graduate from high school in 2015

Dentist rate = number of dentists per 100,000 population in 2015

*North Carolina Department of Health and Human Services variable*

(https://publichealth.nc.gov/oralhealth/services/safetynetclinics.htm)

FQHC with Dental in County = indicator variable for presence of a Federally Qualified Health Center (FQHC) that provides dental services located in the county

In general, non-metro counties have a higher percent of counties classified as Farming/Recreational, classified as places with Persistent Poverty, more Child Abuse Reporting events, a higher Infant Mortality rate, worse rank for Length of Life among counties, and both a lower number of Dentists per population and a lower High School Graduation Rate.

## Regression results

Our multivariate regression results are presented in Table 3. To highlight differences between metro and non-metro counties we estimated the same model for those two subpopulations.

**Table 3:** Multivariate Regression Assessing Determinants of Percent Utilization Rate Among MPW Eligible Women (Dependent variable) Calculated from Pooled Sample During Years 2014–2016, in North Carolina Counties, by Metro vs. Non-metro classification

	Non-metro Counties		Metro Counties		Full Sample	
Variable	Coeff	95% Confidence Interval	Coeff	95% Confidence Interval	Coeff	95% Confidence Interval
Intercept	-18.26	-37.49, 0.97	-12.97	-29.16, 3.23	-16.26	-28.40, -4.12
**Group 1 Predisposing Factors**						
Farming/Recreation	1.53	-1.37, 4.44	-0.002	-2.33, 2.32	1.61**	0.03, 3.20
Persistent Poverty	-4.29**	-7.38, -1.22	-0.81	-6.22, 4.59	-3.11**	-5.46, -0.76
High School Grad Rate	0.20*	-0.02, 0.43	0.18*	-0.009, 0.37	0.19**	0.04, 0.33
**Group 2 County Well-being (need)**						
Abuse reporting past year	0.003	-0.05, 0.05	0.03	-0.02, 0.08	0.01	-0.02, 0.05
Change in Abuse reporting	0.16***	0.08, 0.25	-0.03	-0.19, 0.13	0.13***	0.07, 0.20
Infant Mortality	0.24**	0.07, 0.42	-0.09	-0.39, 0.22	0.18**	0.04, 0.32
Length of Life County Rank	0.05**	0.01, 0.09	0.03	-0.01, 0.07	0.04**	0.01, 0.07
Poor Mental Health Days	1.26**	0.15, 2.38	1.03*	-0.17, 2.23	1.35***	0.59, 2.11
**Group 3 Dental Health Services**						
Dentists per 100,000 pop	0.05	-0.02, 0.12	0.02	-0.008, 0.05	0.03**	0.004, 0.06
FQHC Dental in County	1.28	-0.86, 3.42	1.31	-0.47, 3.09	1.28*	-0.09, 2.65
*R*^*2*^	0.63		0.43		0.51	
Adj *R*^*2*^	0.54		0.25		0.45	
*N*	50		43		93	

Note: * *P* < .10; ** *P* < .05; *** *P* < .001.

Notes on Variables:

*County Codes from US Department of Agriculture (USDA)*

RUCC is the Rural Urban Continuum Code system.^8^ Codes range from 1 to 9. This is a classification scheme developed by the USDA. It attempts to arrange counties on a continuum from most metro to least metro based on population density and proximity of non-metro counties to urban areas

County Typology Codes^9^ for Persistent Poverty, Farming, Recreation

Persistent Poverty is a county-level designation that identifies counties where at least 20 percent of the population is at or below the Federal Poverty Level in each of the census years of 1980, 1990, 2000 and the American Community Survey of 2013. In North Carolina, 10 counties are designated as areas of Persistent Poverty. Persistent Poverty is often a marker that alerts providers of direct and indirect services to the increased vulnerability of the population especially low educational attainment and poor health outcomes

Farming/Recreation is derived to represent counties that were designated as either Farming or Recreation. Farming is the Farm-dependent county indicator, where 0 = no 1 = yes. Farming accounted for at 25% or more of the county’s earnings or 16% or more of the employment averaged over 2010–2012

Recreation defines counties (0 = no 1 = yes) based on computation from three data sources: (1) Percentage of wage and salary employment in entertainment and recreation, accommodations, eating and drinking places, and real estate as a percentage of all employment reported by the Bureau of Economic Analysis; (2) Percentage of total personal income reported for these same categories by the Bureau of Economic Analysis; and (3) Percentage of vacant housing units intended for seasonal or occasional use reported in the 2010 Census. The three variables measuring employment, earnings, and seasonal housing were converted to z-scores and combined into a weighted index (weights of 0.3 were assigned to income and employment and 0.4 to seasonal housing) to reflect recreational activity. Counties with index scores of 0.67 or higher were regarded as recreation counties. Seasonal housing was given a higher weight because in some areas employment and income may not reflect recreational activity because of the seasonality. The comparison group was all other counties and these were designated as either Manufacturing, Government, or Non-specialized

*Variables from NC Child (*http://www.ncchild.org/*)*

Abuse and Neglect claims in 2015 and 2016. The rate per 1000 of children under age 18 who were assessed for abuse or neglect

Infant Mortality in 2015. This represents the number of infant deaths per 1000 babies born alive

*North Carolina County Health Rankings 2015 variables* (http://www.countyhealthrankings.org/rankings/data/NC)

Length of Life – County rank among counties in North Carolina (from 1 to 100, with 100 being worst) for age-adjusted Years of Potential Life Lost before age 75, calculated from 2010–2012 Mortality files National Center for Health Statistics

Quality of Life - from BRFSS 2006–2012, measured as county-level average number of mentally unhealthy days (poor mental health days) in the past 30 days (age-adjusted). Poor mental health days ranged from 2.0 to 6.2 days per month, median of 3.6

High School Graduation Rate = (percent) number students that graduated divided by the number of students expected to graduate from high school in 2015

Dentist rate = number of dentists per 100,000 population in 2015

*North Carolina Department of Health and Human Services variable*

(https://publichealth.nc.gov/oralhealth/services/safetynetclinics.htm)

FQHC with Dental in County = indicator variable for presence of a Federally Qualified Health Center (FQHC) that provides dental services located in the county

The approach is validated by the fact that even though the R-square in full model is 0.488, the explained variation in the non-metro model is 0.618 versus metro with 0.390. Therefore, we conclude that metro versus non-metro areas may be influenced by two different processes, where non-metro populations have less random variation and thus any policy interventions based on our independent variables may be more effective.

The variables for Pre-disposing factors (Group 1) have expected signs and magnitudes with notable differences between metro versus non-metro counties. Farming/Recreation classification only matters for the non-metro counties and same is true for the Persistent Poverty indicator (it is notable that in metro counties those coefficients are effectively zero). High School Graduation rate appears to have slightly more explanatory power in the metro counties.

Factors that represent county-level need are measures of well-being that are relevant for the maternal-child population (Group 2). These factors present very interesting differences between two subpopulations. The variable Change in Abuse Reporting, which in combination with Abuse Reporting Past Year, provides an indication of how the dynamics of abuse reporting affects PUR while holding levels of Abuse Reporting the same. It only matters for the non-metro counties. The same is true for the Infant Mortality variables. On the other hand, Length of Life Ranking and Poor Mental Health Days appear to be important in both models.

The indicators for Dental Health Services (Group 3) show that the impact of the Dentist per population variable appears to be larger in non-metro areas but since it is not statistically significant and the confidence intervals overlap, we cannot determine in which subpopulation it matters more. Whether a county has an FQHC that provides dental services has a similar positive impact in all three models.

The non-metro model shows some strong relationships. Counties with dominant Farming/Recreation economies have a PUR that is higher by about 1.5 percentage points, compared to counties classified otherwise. Counties characterized by Persistent Poverty have PUR that is 4.3 percentages points lower than average which is relatively large since the mean PUR in our sample is about 9.3. A Dentist per population coefficient of 0.05 means that having one more dentist per 100,000 population improves PUR by about 0.05 percentage points. To put this into perspective, if one would double the number of dentists in a non-metro county of 150,000 population from 30 to 60 dentists (which seems unlikely) this would improve utilization by about 1 percentage point. The High School Graduation Rate coefficient is 0.20 which means that the improvement between the worst (68%) and the best county (92.5%) in North Carolina would amount to an improvement in PUR by about 5 percentage points- which is substantial.

## Discussion

Utilization of dental services in the MPW program was low with a median of 8.5 percent of pregnant women having any dental service. There was a broad range across counties. The lowest utilization rate was 1 percent and the highest was 26 percent. Patterns in the dental utilization rate were different when counties were separated as non-metro versus metro areas. In the non-metro sample, community-level patterns were stronger which suggests that non-metro counties maybe influenced more directly from policy changes that leverage community-level factors. Since dental utilization is low statewide, statewide policy changes to improve dental utilization should focus on enhancing the dental benefit to make it easier to use. For example, a first step could be to extend the window of eligibility to 24 months after the birth of the child. Recent evidence shows that improving oral health in pregnant women will improve the child’s oral health [15]. A 24-month window of eligibility would strengthen the value of the MPW program to these communities.

Some of our findings are consistent with other studies that look at dental utilization by rural classifications. These include the lack of a strong relationship between number of dentists in a community and utilization of dental services. Supply and demand in Appalachia counties was not uniform and required spatial analysis techniques to understand utilization of dental services [16]. Patterns suggested that residents may be travelling outside their county of residence to obtain dental services. Comparisons between a multidisciplinary model as used by the Indian Health Service (IHS), showed greater difficulty in making referrals to dentists in non-IHS clinics [17]. This finding is consistent with the concern that pregnant women who are referred for dental care may have more difficulty finding a dentist, especially in rural areas, if there is no care coordination.

In our data for North Carolina, dental service utilization was related to number dentists available, however, not in a consistently robust manner. Four counties with zero dentists had a wide range of dental service utilization and counties classified as most “rural” (i.e., RUCC = 9) had both the highest utilization and the lowest utilization of all counties statewide. Counties with Persistent Poverty had poor utilization and counties classified as Farming or Recreational (versus Non-Specialized, Manufacturing, and/or Government) had better utilization, especially when comparisons included both metro and non-metro counties.

Some of the associations observed may be artifacts or oddities in the county-level measures. These somewhat counter-intuitive observations may signal the existence of other unmeasured factors. For example, increased reporting of child abuse and neglect may reflect an increased awareness of its importance in a community and not necessarily an increase in the prevalence, simply better reporting [18]. However, taken together, the data describe better levels of utilization where some degree of social adversity or negativity exists. We found that counties that had an increase in the number of claims for child abuse and neglect had higher levels of utilization of dental services among women in the MPW program. Similarly, communities with more infant mortality and worse ranking in terms of years of life lost due to premature death had better levels of utilization. It is unclear whether dental needs are greatest in these same communities. Further examination of these factors is needed.

It is interesting to speculate on the measures from the County Health Rankings file that were statistically important in the final model. Both Quality of Life and Length of Life measured at the county level over the period 2006–2012 for number of poor mental health days – a Quality of Life measure - and 2010–2012 for county ranking in number of years of potential life lost before age 75 – a Length of Life measure - represent time periods that precede the time period for the PUR variable in the model (i.e., 2014–2016). Further research should explore whether this lag is meaningful in the dynamics of community social interactions around efforts to improve health outcomes in general and health outcomes for pregnant women specifically.

Infant mortality has been identified as a marker for society’s need to address “access to health care, adequate nutrition, a healthy psychosocial and physical environment, and sufficient income to prevent the adverse consequences of poverty”[19]. One hypothesis for further study is whether these counties with better dental utilization are places that have formed coalitions or community partnerships to address these issues that are part of the constellation of concerns centered around infant mortality. It may be that the data are reflecting markers for communities that have formed partnerships across sectors to address child abuse and neglect and/or infant mortality. Perhaps these communities are places where better support systems are in place for low income pregnant women and this is contributing to better utilization of dental services in the MPW program.

Policy initiatives at the state level may also explain these observed patterns, but more information is needed to go beyond what these contextual indicators highlight. Such policy initiatives include efforts that began in 2013 to implement the Essentials for Childhood Framework to reduce child maltreatment [20] and in 2011 selection of Infant Mortality and Number of Poor Mental Health days as objectives in the State Health Plan, Healthy North Carolina 2020 (HNC2020)[21]. Also, a number of HNC2020 objectives target early loss of life (suicide, homicide, traffic fatalities under the influence of alcohol) that would contribute to a county’s rank for Length of Life. As a call to action, these policy initiatives may bring communities together to address gaps and serve to enhance resilience and this may have had an unanticipated effect on increased utilization of dental services by low income pregnant women.

Ellis and Dietz (2017) have proposed a model for articulating how communities can actively participate in cross-sector efforts to support community resilience. The concept of community resilience [22] identifies a role for adversity and social strain to stimulate community capacities for collective action. Collective action is central to the model where community partners play important roles in care delivery [23]. From this perspective, we can hypothesize that counties with poor performance on metrics for child abuse/neglect, infant mortality, and number of poor mental health days took steps toward collective action around these issues and those collective actions had the collateral effect of improving the ability of a low income pregnant woman to receive dental care.

In the model [24], two key components - Cross-sector Partners and an Engaged Community - may be represented by the community-clinical infrastructure that makes up the Pregnancy Medical Home initiative. The initiative has been a partnership with Community Care of North Carolina (CCNC) and the state’s Medicaid program that began in 2011. It builds on work to address infant mortality that has been an area of emphasis for North Carolina since the early 1990’s. Future research should examine the patient-level and provider-level system dynamics [25] that are linked to services provided by CCNC care coordinators to leverage the Pregnancy Medical Home model to more explicitly address oral health for pregnant women. Further work can build on the progress that has been initiated to help pregnant women get dental services using an academic, interprofessional approach [26]. Pilot interventions have proven successful for improving oral health care service delivery [27]. Additional, policy opportunities may arise if discussions can be framed around community-level benefits though a systems dynamic approach [25].

Co-production provides an alternative economic framework for conceptualizing service delivery involving diverse stakeholders in the public sector [28]. In health care service delivery, this framework expands the traditional doctor-patient dyad with an enhanced model that includes community stakeholders and support systems to add value to the process [29, 30]. This is in contrast to the traditional model for utilization of dental services in underserved areas [31]. The traditional model links low utilization of dental services to dentist availability, utilization costs, and population preferences for dental care. It reflects a dentist-centered model of care that is prone to market failure, given the low reimbursement rate for Medicaid plans and the challenges of low health literacy in the underserved communities. Further work is needed to test hypotheses concerning how co-production can help frame a value proposition that encompasses community stakeholders to improve dental service utilization by low income pregnant women.

Limitations.

As an ecologic study, observed patterns may not be present at the level of the individual. Independent variables are measured at various years, sometimes not even in the range 2014–2016. However, since each mother-birth dyad is independent of each other between years, even if it relates to the same woman, and most independent variables are relatively stable over the years, we anticipate that differences in period measurements would have only a small impact on our results. Our sample is only 100 counties and statistical power is limited. Nevertheless, our results should be treated as a first step toward a more definitive study.

## Conclusions

Predisposing factors among counties such as low high school graduation rates and having Persistent Poverty were associated with low dental service utilization by pregnant women on Medicaid. These factors may compound the effects of material and social disadvantage over time to create a culture of deprivation. Collective action around infant mortality and child well-being may improve dental utilization. A systems dynamic approach that engages community stakeholders across multiple sectors may help shift to a culture of health for low income families.

## Data Availability

The data that support the findings of this study are available from the agencies listed in the acknowledgements section. The datasets generated and analyzed during the current study are available from the corresponding author on reasonable request.

## Abbreviations

CCNC: Community Care of North Carolina
FQHC: Federally Qualified Health Center
HNC2020: Healthy North Carolina 2020
MPW: Medicaid for Pregnant Women program
NCIOM: North Carolina Institute of Medicine
PCMH: Patient-Centered Medical Home
PUR: Percent Utilization Rate for any dental service by eligible enrollees in the Medicaid for Pregnant Women program in North Carolina
RUCC: Rural Urban Continuum Code
SACIM: Secretary’s Advisory Committee on Infant Mortality
